# Lack of Responsiveness during the Onset and Offset of Sevoflurane Anesthesia Is Associated with Decreased Awake-Alpha Oscillation Power

**DOI:** 10.3389/fnsys.2017.00038

**Published:** 2017-05-30

**Authors:** Kara J. Pavone, Lijuan Su, Lei Gao, Ersne Eromo, Rafael Vazquez, James Rhee, Lauren E. Hobbs, Reine Ibala, Gizem Demircioglu, Patrick L. Purdon, Emery N. Brown, Oluwaseun Akeju

**Affiliations:** ^1^Department of Anesthesia, Critical Care and Pain Medicine, Massachusetts General Hospital, Harvard Medical SchoolBoston, MA, United States; ^2^School of Nursing, University of PennsylvaniaPhiladelphia, PA, United States; ^3^Department of Computer Science, Zhejiang UniversityHangzhou, China; ^4^Harvard-Massachusetts Institute of Technology Division of Health Sciences and Technology, Massachusetts Institute of TechnologyCambridge, MA, United States

**Keywords:** awake-alpha oscillations, loss of consciousness, recovery of consciousness sevoflurane, sedation, general anesthesia

## Abstract

Anesthetic drugs are typically administered to induce altered states of arousal that range from sedation to general anesthesia (GA). Systems neuroscience studies are currently being used to investigate the neural circuit mechanisms of anesthesia-induced altered arousal states. These studies suggest that by disrupting the oscillatory dynamics that are associated with arousal states, anesthesia-induced oscillations are a putative mechanism through which anesthetic drugs produce altered states of arousal. However, an empirical clinical observation is that even at relatively stable anesthetic doses, patients are sometimes intermittently responsive to verbal commands during states of light sedation. During these periods, prominent anesthesia-induced neural oscillations such as slow-delta (0.1–4 Hz) oscillations are notably absent. Neural correlates of intermittent responsiveness during light sedation have been insufficiently investigated. A principled understanding of the neural correlates of intermittent responsiveness may fundamentally advance our understanding of neural dynamics that are essential for maintaining arousal states, and how they are disrupted by anesthetics. Therefore, we performed a high-density (128 channels) electroencephalogram (EEG) study (*n* = 8) of sevoflurane-induced altered arousal in healthy volunteers. We administered temporally precise behavioral stimuli every 5 s to assess responsiveness. Here, we show that decreased eyes-closed, awake-alpha (8–12 Hz) oscillation power is associated with lack of responsiveness during sevoflurane effect-onset and -offset. We also show that anteriorization—the transition from occipitally dominant awake-alpha oscillations to frontally dominant anesthesia induced-alpha oscillations—is not a binary phenomenon. Rather, we suggest that periods, which were defined by lack of responsiveness, represent an intermediate brain state. We conclude that awake-alpha oscillation, previously thought to be an idling rhythm, is associated with responsiveness to behavioral stimuli.

## Introduction

Anesthetic drugs are typically administered to induce altered states of arousal that range from sedation to general anesthesia (GA), a reversible state comprised of unconsciousness, amnesia, analgesia and immobility with maintenance of physiological stability (Brown et al., 2010). Studies of anesthesia-induced neural oscillations provide a framework that currently guide investigations of the neural circuit mechanisms of anesthesia-induced altered arousal states (McCarthy et al., 2008; Ching et al., 2010; Murphy et al., 2011; Supp et al., 2011; Boly et al., 2012; Vijayan and Kopell, 2012; Lee et al., 2013; Ní Mhuircheartaigh et al., 2013; Purdon et al., 2013; Vijayan et al., 2013; Hashemi et al., 2015). These studies suggest that by disrupting the oscillatory dynamics that are associated with arousal states, anesthesia-induced oscillations are a putative mechanism through which anesthetic drugs produce altered states of arousal (McCarthy et al., 2008; Ching et al., 2010; Murphy et al., 2011; Boly et al., 2012; Vijayan and Kopell, 2012; Lee et al., 2013; Ní Mhuircheartaigh et al., 2013; Purdon et al., 2013; Vijayan et al., 2013; Hashemi et al., 2015). Further, each anesthetic drug class has been shown to produce distinct neural oscillations that can be related to the drug’s mechanism of action (Purdon et al., 2015).

Extensive work has been done to relate the neural oscillations induced by propofol (2,6-di-isopropylphenol), an intravenous anesthetic that primarily potentiates γ-Aminobutyric acid A (GABA_A_) receptors, to its neural circuit mechanisms (Ching et al., 2010; Murphy et al., 2011; Supp et al., 2011; Boly et al., 2012; Lewis et al., 2012; Purdon et al., 2013; Vijayan et al., 2013; Hashemi et al., 2015). Beta (13–33 Hz) oscillations are associated with propofol sedation (Purdon et al., 2013, 2015), while large amplitude slow-delta (0.1–4 Hz) and coherent frontal alpha (8–12 Hz) are associated with propofol GA (Murphy et al., 2011; Lewis et al., 2012; Ní Mhuircheartaigh et al., 2013; Purdon et al., 2013; Akeju et al., 2014a,b; Hashemi et al., 2015). Propofol beta oscillations likely result from a modest increase in GABA_A_ decay-time and conductance, which causes low threshold spiking (LTS) interneuron antisynchrony that patterns pyramidal cell spiking into a beta rhythm (McCarthy et al., 2008). Potentiation of the GABA synaptic currents also causes a reduction in a slow potassium membrane current that further increases LTS interneuron excitability. Additional increases in GABA_A_ decay-time and conductance results in cortical alpha oscillatory dynamics and enhanced rebound spiking of thalamic relay cells, which strengthen the intrinsic alpha oscillatory dynamics of the thalamus (Ching et al., 2010; Vijayan and Kopell, 2012; Vijayan et al., 2013). The net result is reciprocal thalamic-cortical alpha oscillation coupling (Ching et al., 2010; Vijayan and Kopell, 2012; Vijayan et al., 2013; Hashemi et al., 2015). Slow oscillations may result from decreased excitatory cortical inputs from brainstem arousal nuclei, and from simultaneous drug action in the thalamus and cortex (Brown et al., 2010; Hashemi et al., 2015).

The neural circuit mechanism of sevoflurane, an anesthetic vapor that is routinely administered to produce sedation or GA, has not been extensively studied. At the molecular level, sevoflurane potentiates GABA_A_, glycine and two-pore potassium channels, and inhibits voltage-gated potassium, N-methyl-D-aspartate (NMDA), muscarinic and nicotinic acetylcholine (ACH), serotonin and α-Amino-3-hydroxy-5-methyl-4-isoxazolepropionic acid (AMPA) channels (Campagna et al., 2003; Rudolph and Antkowiak, 2004; Franks, 2008). Sevoflurane sedation and GA states are also associated with highly structured oscillations that disrupt the oscillatory dynamics necessary for conscious processing. Like propofol, beta (13–33 Hz) oscillations are associated with sevoflurane sedation (Blain-Moraes et al., 2015; Kaskinoro et al., 2015; Purdon et al., 2015), and large amplitude slow-delta (0.1–4 Hz) and coherent frontal alpha (8–12 Hz) are associated with sevoflurane GA (Akeju et al., 2014b). These similarities suggest that GABA_A_ circuit mechanisms also produce sevoflurane-induced altered states of arousal.

At a relatively stable anesthetic dose, an empirical clinical observation is that patients may sometimes be intermittently responsive to verbal commands during states of light sedation. Although large amplitude slow-delta and coherent frontal alpha oscillatory dynamics during sevoflurane-induced moderate-deep sedation and GA have been demonstrated, it is not clear whether these dynamics explain intermittent responsiveness. This is because a study of propofol electroencephalogram (EEG) oscillations found that these oscillatory dynamics were notably absent during light sedation (Akeju et al., 2014a). Thus, correlates of intermittent responsiveness during anesthesia effect-onset and -offset, brain states that are typically associated with beta oscillations, require further exploration. A principled understanding of these neural correlates may fundamentally advance our understanding of neural dynamics that are essential for maintaining arousal states, and how they are disrupted by anesthetics.

Therefore, we performed a high-density (128 channels) EEG study of sevoflurane-induced altered arousal with temporally-precise behavioral stimuli that were administered every 5 s. The prominent oscillatory dynamic during the awake, eyes-closed state is alpha oscillations. We refer to this dynamic as *awake-alpha oscillations* to distinguish it from *sevoflurane-induced alpha oscillations*. We found that lack of responsiveness during sevoflurane anesthesia effect-onset and -offset is associated with the loss of awake-alpha oscillations. We also found that anteriorization, the transition from occipital awake-alpha oscillations to frontal sevoflurane-induced alpha oscillations, is not a binary phenomenon, and that periods, which were defined by lack of responsiveness, represent an intermediate brain state.

## Materials and Methods

### Volunteer Selection

Volunteers were recruited for this study between January 2015 and May 2015 via an institutional email broadcast that alerts employees within our healthcare system to ongoing studies. Prior to enrollment, potential volunteers underwent a complete medical history, as well as standard pre-anesthesia assessments. Inclusion criteria consisted of; normal body weight and habitus (BMI ≤ 30), non-smoker, and American Society of Anesthesiology Physical Status I with Mallampati Class I airway anatomy. We excluded volunteers who were pregnant, or had a history of obstructive sleep apnea, gastroesophageal reflux, cardiac conduction abnormalities, asthma, epilepsy, history of problems with anesthesia, family history of problems with anesthesia, history of drug use, and any neurologic or psychiatric history. Other procedures included urine toxicology screen to rule out prohibited drug use, serum pregnancy test for females, and an electrocardiogram to rule out cardiac conduction deficits. Written informed consent was obtained from a total of eight (4 males) healthy volunteers, 24 (SD ± 2.6) years of age, during the screening visit. Mean weight was 73.7 (23.1) kg and BMI was 24.9 (4.1) kg/m^2^.

### Experimental Protocol and Recording

This study was performed in the Carl Rosow Clinical Research Center at the Massachusetts General Hospital. Prior to the start of the study volunteers were required to take nothing by mouth for at least 8 h. The morning of the study, a urine toxicology screen was performed to rule out the use of prohibited substances. Additionally, a urine pregnancy test was performed to rule out pregnancy in all female study volunteers. We monitored the heart rate with electrocardiogram, oxygen saturation with pulse oximetry, respiration and expired carbon dioxide with capnography, and blood pressure with a standard non-invasive cuff. A peripheral intravenous line was placed for the potential administration of rescue medications. Prior to anesthetic delivery, adequate face-mask seal and comfort was confirmed for every volunteer.

**Figure 1:**
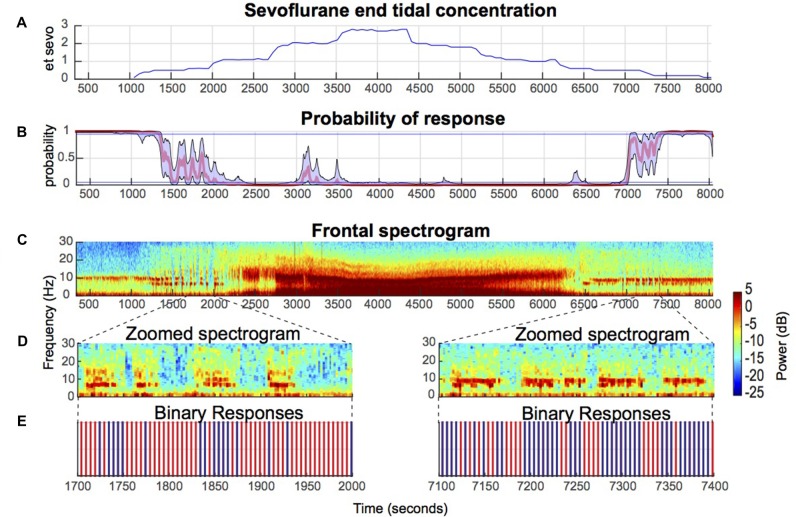
Illustrative electroencephalogram (EEG) spectrogram of a healthy volunteer during induction and emergence from sevoflurane-induced unconsciousness. **(A)** End tidal sevoflurane concentration during induction and emergence. **(B)** Behavioral stimuli response-probability curve corresponding to **(A)**. **(C)** EEG spectrograms from a frontal channel (approximately Fz) corresponding to **(A,B)** above show that sevoflurane-induced oscillatory dynamics are closely associated with altered arousal. **(D)** Zoomed in spectrogram during effect-onset and -offset. **(E)** Binary response demonstrates that loss of awake-alpha is correlated with lack of response (red) to behavioral stimuli.

Volunteers were instructed to close their eyes throughout the study to avoid eye-blink artifacts in the EEG. Eye closure facilitates distinguishing between normal awake, eyes-closed occipital alpha oscillations and anesthetic-induced oscillations. Volunteers received sevoflurane anesthesia, delivered via a Dräger Fabius Tiro (Telford, PA, USA) anesthesia machine, in high-flow oxygen/air admixture through a secured facemask to achieve the following end tidal concentrations: 0.5, 1.2 and 2.8% (Figure 1A). The inspired sevoflurane concentration was adjusted until the desired end tidal sevoflurane concentration was achieved. Each sevoflurane end tidal concentration target was maintained for 12 min. In addition to the study team, a board certified anesthesiologist, not involved in data acquisition or other study functions, monitored and cared for each volunteer. Standard airway maneuvers (i.e., chin lift, jaw thrust) were used to maintain normal oxygenation and ventilation. The airway was not instrumented in any volunteer.

We recorded the EEG using the Waveguard system (ANT Neuro, Netherlands) with a sampling frequency of 2056 Hz, average EEG referencing, and electrode impedances of <5 kΩ Data were acquired with the equidistant, hexagonal 128-channel EEG cap (ANT Neuro, Netherlands). Data were down-sampled to 250 Hz and interpolated (bad channels) using ASA-Lab software (ANT Neuro, Netherlands). Continuous EEG data files were saved and stored for off-line processing. During the experiment, the volunteers were instructed to press a button when auditory or sensory stimuli were presented. The auditory tasks consisted of a prerecorded verbal command to press a button, and a train of clicks, which were presented at random every 5 s. After the presentation of 11 auditory tasks (55-s), a sensory stimulus—a cuff pain device attached to the gastrocnemius muscle and titrated to 7/10 pain—was presented. All behavioral stimuli were time locked to the EEG data.

### Statistical Power Analysis

An* a priori* sample size calculation was not performed for this pilot study. The sample size was based on a similar neurophysiological study of sevoflurane GA (Blain-Moraes et al., 2015).

### Behavioral Analysis

We used a Bayesian state-space algorithm to estimate and relate to the EEG, each volunteer’s response probability [Pr (Response) curve] from the binary responses to the behavioral stimuli (Purdon et al., 2013; Wong et al., 2014). We defined awake as Pr (Response) > 0.95, and unconsciousness (deep sedation, GA) as Pr (Response) < 0.05. A 60-s artifact-free epoch with Pr (Response) > 0.95, preceding the administration of sevoflurane was chosen for the baseline-awake, eyes-closed period. A 60-s, artifact-free epoch with a Pr (Response) < 0.05 was chosen for the GA state, approximately 6 min after the end tidal concentration of sevoflurane registered −2% (during drug up titration). We defined sevoflurane effect-onset as the time period during the induction of sevoflurane anesthesia when the probability of response Pr (Response) was between 0.05 and 0.95 for a minimum of 5 min. We defined sevoflurane effect-offset as the time period during emergence from sevoflurane anesthesia when the Pr (Response) was between 0.05 and 0.95. During the effect-onset and -offset periods, study volunteers responded intermittently to all behavioral stimuli.

### Spectral Analysis

Multitaper spectral estimates were computed as implemented in the Chronux toolbox. We derived frontal and occipital electrodes to better reflect EEG dynamics unique to frontal and occipital regions. To derive these electrodes, we averaged seven frontal electrodes that approximated the locations of Fz and Oz. The parameters for the multitaper spectral analysis were: window length *T* = 2.5 s with no overlap, time-bandwidth product TW = 3, number of tapers *K* = 5, and spectral resolution = 2.4 Hz. We assumed signal stationarity beginning 2.5 s before stimulus presentation and ending 2.5 s after stimulus presentation. Thus, 5 s of data were extracted for each behavioral stimulus. Multitaper spectral estimates and median spectral estimates for both response and no-response were computed at the individual level. Median 5 s spectral estimates were also computed for the baseline-awake and GA epochs. Group-level spectrograms were computed by taking the median spectral estimates across all volunteers.

### Topographic Analysis

Scalp power distributions of the alpha frequency band were computed using interpolation of the electrode montage with the topoplot function in EEGLab (Delorme and Makeig, 2004). We computed group-averaged spectra by taking the mean of the group-level spectrograms across time and then averaged them over the frequency band of interest for each electrode.

### Statistical Analysis

To assess statistical significance for the difference in spectra at each frequency, we computed the 99% confidence interval of the median difference between groups by using an empirical bootstrap approach. We resampled spectral estimates for each non-overlapping window to obtain replicates of the estimates for each volunteer, and took the median value across volunteers for each group. We took the difference between two median estimates, repeated this 1000 times and calculated the 99% confidence interval of the median difference at each frequency. Each spectrum in this manuscript represents the median of the distribution of median spectra across volunteers, with confidence intervals drawn from the distribution of median spectra. For frequencies f > 2W, the null hypothesis was rejected only if the confidence interval of the median difference at each frequency exceeded the significance threshold over a contiguous frequency range ≥ 2W. For frequencies 0 ≤ f ≤ 2W, to account for the properties of multitaper spectral estimates at frequencies close to zero, the null hypothesis was rejected only if the confidence interval of the median difference at each frequency exceeded the significance threshold over a contiguous frequency range from 0 to max (f, W) ≤ W.

## Results

No serious adverse events occurred during this study.

### Sevoflurane Anesthesia Effect-Onset and -Offset Were Defined by Intermittent Responses to Behavioral Stimuli

Consistent with a previous study of propofol-induced anesthesia, during sevoflurane-induced anesthesia onset and offset, the probability of response did not linearly transition from 1 to 0, and vice versa (Figure 1B). Rather, increases and decreases to the response probability, which reflect response and no-response state changes, were observed (Figure 1B). Also consistent with a previous study of propofol-induced anesthesia, during up titration of the end tidal sevoflurane concentration from 1% (moderate-deep sedation) to 2% (GA)—two brain states with Pr (Response) <0.05—beta oscillations that transition to alpha oscillations were observed (Figures 1A–C). This finding was conserved during down titration of the end tidal sevoflurane concentration from 2% to 1% (Figures 1A–C). Beta oscillations were also present during effect-onset and -offset periods (Figures 1C,D). However, unlike the moderate-deep sedation beta oscillations, the effect-onset and -offset beta oscillations were not tightly constrained in the beta frequency band (Figures 1C,D). Importantly, loss of the awake-alpha oscillations was correlated with lack of response to behavioral stimuli during sevoflurane effect-onset and -offset (Figures 1D,E).

### Scalp Spatiotemporal Representation of Awake-Alpha Oscillations Demonstrated Decreased Power during No-Response Periods

**Figure 2:**
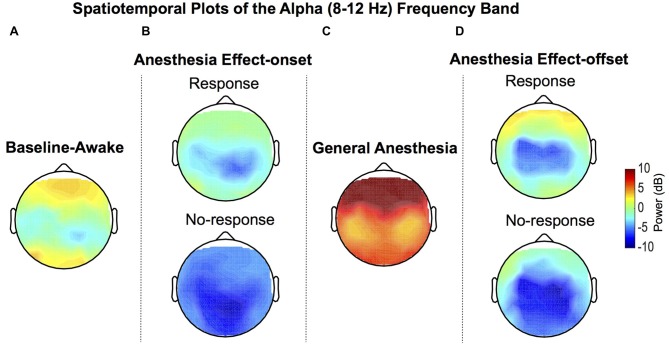
Group-level spatial distribution of alpha power. **(A)** Alpha oscillations can be observed during the baseline-awake state. **(B)** Lack of response during sevoflurane anesthesia effect-onset was associated with decreased awake-alpha oscillations compared to response. **(C)** Large amplitude frontal alpha-oscillations were observed with sevoflurane general anesthesia (GA). **(D)** Lack of response during sevoflurane anesthesia effect-offset was associated with decreased awake-alpha oscillations compared to response.

Group-median topographic plots of power for the alpha frequency band were computed for the baseline-awake, effect-onset, GA and effect-offset states (Figure 2). Expectedly, the well-described awake occipital alpha oscillation was prominent during the baseline-awake state because study volunteers were lying awake with their eyes closed (Figure 2A). During sevoflurane effect-onset increased alpha oscillation power was evident in frontal and occipital regions during the response vs. no-response periods (Figure 2B). GA-induced alpha oscillations, a dynamic that is distinct from the awake-alpha oscillations, were higher in power and localized to frontal brain regions (Figure 2C). During sevoflurane effect-offset, increased alpha oscillation power was also evident in frontal and occipital regions during the response period, compared to the no-response period (Figure 2D).

### Lack of Responsiveness during Sevoflurane Anesthesia Effect-Onset Was Associated with Significantly Reduced Awake-Alpha Powers

**Figure 3:**
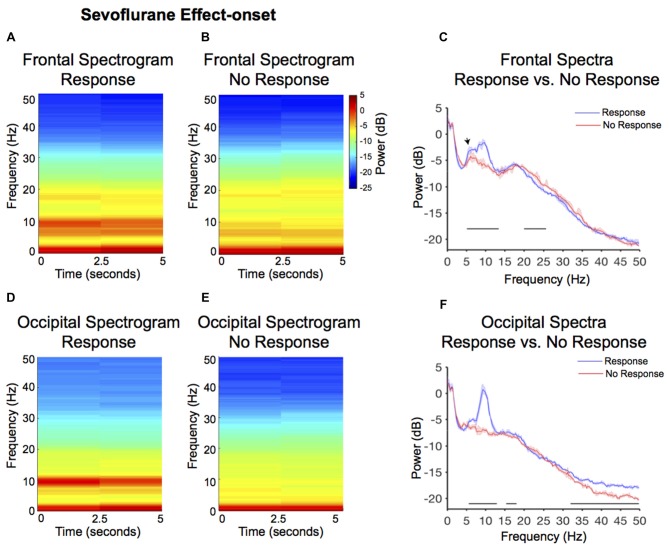
Spectral comparison of response vs. no-response curves during anesthesia effect-onset. **(A,B)** Median frontal spectrograms of response and no-response (*n* = 8). **(C)** Overlay of median response frontal spectrum (blue), and median no-response frontal spectrum (red). Bootstrapped median spectra are presented, and the shaded regions represent the 99% confidence interval for the uncertainty around each median spectrum. We observed differences in power among frequency bands within response and no-response spectra (Table 1). Black lines represent statistically significant frequencies. **(D,E)** Median occipital spectrograms of response and no-response (*n* = 8). **(F)** Overlay of median response occipital spectrum (blue), and median no response occipital spectrum (red), with shaded regions showing the 99% confidence interval for uncertainty around each bootstrapped median spectrum. We observed differences in power among frequency bands within response and no-response spectra (Table 1). Black lines represent statistically significant frequencies.

Larger alpha and theta (4–8 Hz) power were observed in the response frontal spectrogram compared to the no-response occipital spectrogram (Figures 3A,B). We evaluated frontal power differences between the response and no-response spectra and found that the no-response spectrum did not exhibit an awake-alpha oscillation peak (Figure 3C). The no-response spectrum exhibited significantly decreased power in theta and alpha frequency bands (Figure 3C, Table 1; 5.1–13.4 Hz), and significantly increased power in the beta frequency band (Figure 3C, Table 1; 20–25.6 Hz). The response spectrum exhibited a distinct theta oscillation peak (Figure 3C; black arrow).

**Table 1:** Significant differences in frequencies.

	Location	Significant frequencies
**Sevoflurane effect-onset**	Frontal	no-resp > resp: 20–25.6 Hz resp > no-resp: 5.1–13.5 Hz
Response vs. no-response	Occipital	no-resp > resp: - resp > no-resp: 5.6–12.9 Hz, 15.4–18.1 Hz, 31.9–50 Hz
**Sevoflurane effect-offset**	Frontal	no-resp > resp: 17.6–30.5 Hz resp > no-resp: 7.1–10.7 Hz, 37.6–50 Hz
Response vs. no-response	Occipital	no-resp > resp: 19.8–23.2 Hz resp > no-resp: 6.6–11 Hz, 31.3–50 Hz
Baseline awake vs. response	Frontal	baseline awake > resp: 2.2–4.4 Hz, 8.8–11.5 Hz resp > baseline awake: 5.6–8.3 Hz, 12.2–34.7 Hz
	Occipital	baseline awake > resp: 1.9–4.4 Hz, 8.8–11.9 Hz, 32.9–50 Hz resp > baseline awake: 13.9–18.8 Hz
Baseline awake vs. general anesthesia	Frontal	baseline awake > GA: 36.9–50 Hz GA > baseline awake: 0.1–32.7 Hz
	Occipital	baseline awake > GA: 27.6–50 Hz GA > baseline awake: 0.1–21 Hz

*GA, General anesthesia; Hz, Hertz; no-resp, No Response; resp, Response*.

Larger alpha and theta power were also observed in the response occipital spectrogram compared to the no-response occipital spectrogram (Figures 3D,E). We evaluated occipital power differences between response and no-response spectra and found that the no-response spectrum did not exhibit an awake-alpha oscillation peak (Figure 3F). The no-response spectrum exhibited significantly decreased power in various frequency bands (Figure 3F, Table 1; 5.6–12.9 Hz, 15.4–18.1 Hz, 31.9–50 Hz). Subject level no-response spectra are presented in Supplementary Figure S1.

### Lack of Responsiveness during Sevoflurane-Anesthesia Effect-Offset Was Associated with Significantly Reduced Relaxed-Wakefulness Alpha Power

**Figure 4:**
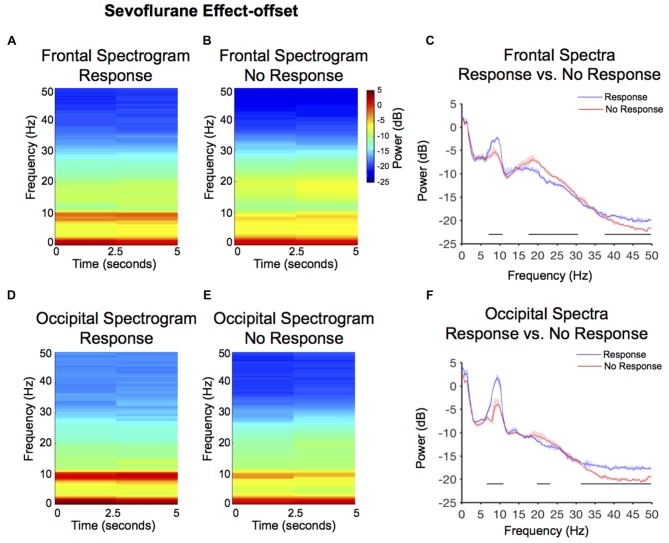
Spectral comparison of response vs. no-response curves during anesthesia effect-offset. **(A,B)** Median frontal spectrograms of response and no-response (*n* = 8). **(C)** Overlay of median response frontal spectrum (blue), and median no-response frontal spectrum (red). Bootstrapped median spectra are presented, and the shaded regions represent the 99% confidence interval for the uncertainty around each median spectrum. We observed differences in power among frequency bands within response and no-response spectra (Table 1). Black lines represent statistically significant frequencies. **(D,E)** Median occipital spectrograms of response and no-response (*n* = 8). **(F)**Overlay of median response occipital spectrum (blue), and median no-response occipital spectrum (red), with shaded regions showing the 99% confidence interval for uncertainty around each bootstrapped median spectrum. We observed differences in power among frequency bands within response and no-response spectra (Table 1). Black lines represent statistically significant frequencies.

Larger alpha power, but not theta power, was observed in the response frontal spectrogram compared to the no-response frontal spectrogram (Figures 4A,B). We evaluated frontal power differences between the response and no-response spectra, and found that the no-response spectrum exhibited significantly decreased power in the alpha and gamma frequency bands (Figure 4C, Table 1; 7.1–10.7 Hz, 37.6–50 Hz), and significantly increased power in the beta frequency band and (Figure 4C, Table 1; 17.6–30.5 Hz).

Larger alpha power was also observed in the response occipital spectrogram compared to the no-response occipital spectrogram (Figures 4D,E). We evaluated occipital power differences between response and no-response spectra and found that although the no-response period exhibited an awake-alpha oscillation peak, it was significantly decreased in power (Figure 4F, Table 1; 6.6–11 Hz). Consistent with the difference seen in occipital effect-onset spectral power (Figure 3F), the no-response spectrum exhibited significantly decreased gamma oscillation power (Figure 4F, Table 1; 31.3–50 Hz).

### Differences in Alpha, Frontal Beta and Occipital Gamma Power Distinguished the Baseline Awake State from the Effect-Onset Responsive State

**Figure 5:**
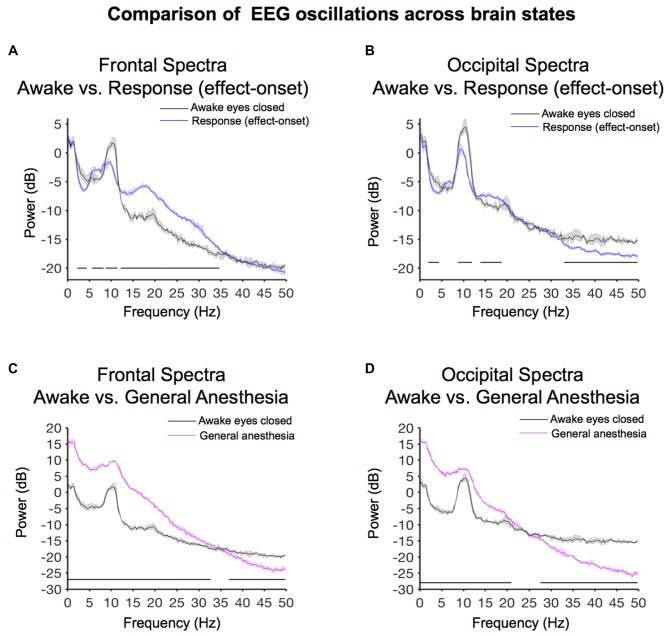
Spectral comparison of baseline-awake vs. altered brain states. **(A)** Overlay of median baseline-awake frontal spectrum (black), and median effect-onset (response) frontal spectrum (blue). Bootstrapped median spectra are presented and the shaded regions represent the 99% confidence interval for the uncertainty around each median spectrum. We observed differences in power among frequency bands (Table 1). Black lines represent statistically significant frequencies. **(B)** Overlay of median baseline-awake occipital spectrum (black), and median effect-onset (response) occipital spectrum (blue), with shaded regions depicting the 99% confidence interval for uncertainty around each bootstrapped median spectrum. We observed differences in power among frequency bands (Table 1). Black lines represent statistically significant frequencies.**(C)** Overlay of median baseline-awake frontal spectrum (black), and median general anesthesia frontal spectrum (magenta). Bootstrapped median spectra are presented and the shaded regions represent the 99% confidence interval for the uncertainty around each median spectrum. We observed differences in power among frequency bands (Table 1). Black lines represent statistically significant frequencies. **(D)** Overlay of median baseline-awake occipital spectrum (green), and median GA occipital spectrum (pink), with shaded regions depicting the 99% confidence interval of uncertainty around each bootstrapped median spectrum. We found differences in power between baseline-awake and effect-onset (response) frequency bands, as well as between baseline-awake and GA frequency bands (frontal response > baseline-awake: 5.9–8.3 Hz, 12.2–36.4 Hz; frontal response < baseline-awake: 2–4.2 Hz, 8.8–11.5 Hz; occipital response > baseline-awake: 14.4–18.3 Hz; occipital response < baseline-awake: 2–5.1 Hz, 8.5–11.2 Hz, 28.3–32.5 Hz, 35.2–49.8 Hz; frontal GA > baseline-awake: 0.1–31.5 Hz; frontal GA < baseline-awake: 37.6–49.8 Hz; occipital GA > baseline-awake: 0.1–19.8 Hz; occipital GA < baseline-awake: 24.9–49.8 Hz).

We evaluated frontal power differences between baseline-awake and sevoflurane effect-onset spectra, to decipher sevoflurane-induced EEG changes associated with the transition to altered arousal states. We found that the baseline-awake spectrum exhibited significantly increased delta and awake-alpha oscillation power (Figure 5A, Table 1; 2.2–4.4 Hz, 8.8–11.5 Hz and decreased theta and beta oscillation power (Figure 5A, Table 1; 5.6–8.3 Hz; 12.2–34.7 Hz).

We evaluated occipital power differences and found that the baseline-awake spectrum exhibited significantly increased awake-alpha oscillation and gamma oscillation power (Figure 5B, Table 1; 8.8–11.9 Hz, 32.9–50 Hz). The response (effect-onset) spectrum exhibited significantly increased beta oscillation power (Figure 5B, Table 1; 13.9–18.8 Hz), and decreased delta oscillation power (Figure 5B, Table 1; 1.9–4.4 Hz). Subject level baseline-awake spectra are presented in Supplementary Figure S1.

### Sevoflurane-General Anesthesia Alpha Oscillations Were Larger in Power than Awake-Alpha Oscillations

We evaluated frontal power spectra differences during sevoflurane GA and the baseline-awake state to study differences between sevoflurane-induced alpha oscillations and awake-alpha oscillations. We found that the peak sevoflurane-induced alpha oscillation power of 9.6 [CI: 9.4 9.9] dB was much larger than awake-alpha oscillation power 1.8 [0.8 2.4] dB. Further, the alpha peak to slow peak ratio of −6.2 dB demonstrates that slow oscillations were the predominant oscillatory dynamic during this state (Figure 5C). This is in contrast to the alpha peak to slow peak ratio of −0.5 dB during the baseline-awake state (Figure 5A). We found that the sevoflurane GA spectrum exhibited significantly increased slow-delta, theta, alpha, beta oscillation power (Figure 5C, Table 1; 0.1–32.7 Hz), and decreased gamma oscillation power (Figure 5C, Table 1; 36.9–50 Hz).

We also evaluated occipital power spectra differences during sevoflurane GA and found that the peak sevoflurane-induced alpha oscillation power of 7.4 [7.3 8] dB was much larger than awake-alpha oscillation power of 4.5 [3.2 5.3] dB. The alpha peak to slow peak ratio of −8.6 dB also demonstrates that slow oscillations were the predominant oscillatory dynamic during this state (Figure 5D). This is in contrast to the alpha peak to slow peak ratio of 1.4 dB during the baseline-awake state (Figure 5B). We also found that the sevoflurane GA spectrum exhibited significantly increased slow-delta, theta, alpha, beta oscillation power (Figure 5D, Table 1; 0.1–21 Hz), and decreased beta and gamma oscillation power (Figure 5D, Table 1; 27.6–50 Hz). Subject level sevoflurane GA spectra are presented in Supplementary Figure S1.

## Discussion

In this investigation, we studied EEG correlates of intermittent responsiveness to behavioral stimuli during sevoflurane anesthesia-induced altered arousal states that clinically correspond to light sedation. Our main findings were that during sevoflurane anesthesia effect-onset, there was an increase in frontal theta and frontal beta power (Figures 5A,B, Table 1). However, decreased awake-alpha power (Figure 3, Table 1; frontal and occipital), increased beta power (Figure 3, frontal and occipital), and decreased gamma power (Figures 3D–F, Table 1; occipital) distinguished when volunteers did not respond to behavioral stimuli, compared to when they responded during sevoflurane anesthesia effect-onset. Similarly, decreased awake-alpha power (Figure 4, Table 1; frontal and occipital), increased beta power (Figure 4, Table 1; frontal and occipital), and decreased gamma power (Figures 4D–F, Table 1; occipital) distinguished when volunteers did not respond to behavioral stimuli, compared to when they responded during sevoflurane anesthesia effect-offset. We contrast the awake-alpha oscillations described above from the sevoflurane GA alpha oscillation by noting that sevoflurane alpha oscillations are frontally dominant and coherent, larger in amplitude, and are associated with large amplitude slow oscillations (Akeju et al., 2014b; Purdon et al., 2015). These functional differences make clear that sevoflurane GA alpha oscillations and awake-alpha oscillations are distinct oscillatory dynamics.

### Awake-Alpha Oscillations Do Not Reflect an Idling Dynamic

Awake-alpha oscillations, which are associated with the “restful with eyes closed” state, were first described in the 1920s (Adrian and Yamagiwa, 1934). Because the power of this oscillatory dynamic was suppressed by visual and tactile somatosensory stimuli, awake-alpha oscillations were proposed to reflect idling of task-irrelevant cortical areas (Adrian and Matthews, 1934; Adrian and Yamagiwa, 1934). More recently, enhanced awake-alpha oscillations described during memory encoding and retrieval tasks suggest that this oscillatory dynamic is associated with higher cognitive processes (Klimesch et al., 2007; Palva and Palva, 2007). In support of this notion, a magnetoencephalogram study has demonstrated that awake-alpha oscillations, in brain regions that are relevant for attention and consciousness, selectively phase lock to somatosensory stimuli that are consciously perceived (Palva et al., 2005). Further, a combined EEG and functional magnetic resonance study has linked alpha phase synchrony to cognitive functions associated with the fronto-parietal network (Sadaghiani et al., 2012). Our finding of decreased awake-alpha power during sevoflurane effect-onset and -offset, when volunteers did not respond to behavioral stimuli, is consistent with a role for awake-alpha oscillations in neuronal processes supporting arousal.

### Sleep Onset Is Associated with Loss of Awake-Alpha Oscillations

Sleep is a naturally occurring state of arousal that is crucial for normal health (Espana and Scammell, 2011; Brown et al., 2012; Schwartz and Kilduff, 2015). Amongst other physiological measurements (electrooculogram, electromyogram), EEG neural oscillations are used to empirically characterize sleep into rapid eye movement (REM) sleep and non-rapid eye movement (NREM) stages 1–3 (N1–N3) sleep (Brown et al., 2012; Prerau et al., 2017). Sleep onsets typically begins with stage N1 sleep, before transitioning to stage N2, stage N3 and REM sleep. N1 sleep is defined by the gross disappearance of awake-alpha oscillations and an increase in slow-delta and theta oscillation power (Brown et al., 2012; Prerau et al., 2017). We note that short bursts of alpha power, which have been suggested to reflect micro-arousals or complete arousals, have also been described during REM sleep (Cantero et al., 1999, 2002). However, it is likely that awake-alpha oscillations do not share functional characteristics with REM sleep alpha bursts. Recently, a high density EEG study that used behavioral stimuli and multi taper spectral estimation techniques to more precisely track EEG dynamics during the sleep onset process, found that loss of awake-alpha oscillations was correlated with a lack of response to behavioral stimuli (Prerau et al., 2014). This finding is consistent with our results. However, decreased beta oscillation power is associated with loss of awake-alpha oscillations during the sleep onset process, while increased beta oscillation power is associated with loss of awake-alpha oscillations during sevoflurane anesthesia effect-onset and -offset.

### Mechanisms to Explain the Awake-Alpha Oscillation

A model has shown that occipital awake-alpha oscillations may be generated through the actions of high-threshold thalamocortical (HTC) neurons (Vijayan and Kopell, 2012). An extension of this model also explains anteriorization of propofol-induced alpha oscillations (Vijayan and Kopell, 2012; Vijayan et al., 2013), and by proxy, sevoflurane. Alpha activity in the occipital component is abolished because a reduction of hyperpolarization-activated cation current (I_H_) conductance silences HTC neurons, the putative generators of occipital alpha (Vijayan et al., 2013). Conversely, propofol-induced frontal alpha oscillations arise from increased GABA_A_ decay-time and conductance that results in cortical alpha oscillatory dynamics and enhanced rebound spiking of thalamic relay cells (Ching et al., 2010; Vijayan et al., 2013). Our present findings suggest that reduction of I_H_ conductance and the associated silencing of HTC neurons occurs much earlier than the reciprocal thalamocortical alpha oscillation feedback loop that is induced by GABAergic inhibition. Thus, anteriorization is not a binary phenomenon, and lighter states of altered arousal (i.e., stage N1 sleep, light sedation) where humans may be roused to wakefulness represent an intermediate brain state that is associated loss of awake-alpha oscillations. This is consistent with a recent report, which showed that anteriorization was not reliably associated with sevoflurane-induced unconsciousness (Blain-Moraes et al., 2015). We suggest that anteriorization of GA alpha oscillations, a neural dynamic that is associated with large amplitude slow-delta oscillations (Lewis et al., 2012; Ní Mhuircheartaigh et al., 2013; Purdon et al., 2013; Vijayan et al., 2013; Akeju et al., 2014a,b, 2016), is indicative of a more profound state of altered arousal.

It is reasonable to conclude that loss of awake-alpha oscillations during the sleep onset process is mediated primarily by subcortical mechanisms. This is because sleep is actively generated in the brainstem, hypothalamus and basal forebrain (Brown et al., 2011, 2012; Espana and Scammell, 2011; Schwartz and Kilduff, 2015; Weber and Dan, 2016). We suggest that decreased awake-alpha oscillations during sevoflurane anesthesia may be mediated by subcortical-cortical mechanisms. This is because when volunteers responded during sevoflurane anesthesia effect-onset (a brain state that supports responsiveness to behavioral stimuli), a theta oscillation peak was coincident with beta oscillations. Abnormal increases in theta power and beta power have been associated with mild deafferentation and disfacilitation of thalamic neurons (Jeanmonod et al., 1996; Llinás et al., 1999, 2005; Hall et al., 2010; Williams et al., 2013). However, we found it interesting that a dynamic, was not associated with responses during sevoflurane anesthesia effect-onset. We suggest that this putative mild deafferentation and disfacilitation dynamic may have occurred prior to sevoflurane effect-offset.

We note that an analysis of human intracranial electrode data acquired during REM sleep recently demonstrated prominent theta-beta network oscillations in the anterior cingulate cortex and the dorsolateral prefrontal cortex (Vijayan et al., 2017). The larger frontal theta and beta power demonstrated in our frontal electrode compared to the occipital electrode suggests that theta-beta dynamics during these two altered states of arousal may be present in common brain structures. However, a more detailed characterization of theta-beta dynamics during these two altered arousal states is clearly necessary. Mechanisms to explain theta oscillations in humans during altered arousal states are yet to be deciphered. However, a lesion study of the precoeruleus, which projects to widespread areas of the brain (i.e., thalamus, hypothalamus, preoptic area) abolished REM sleep theta oscillations in a rodent model (Lu et al., 2006). Thus, theta-beta network oscillations may represent a subcortical-cortical network dynamic.

### Reconciling Differences between Response Spectra during Effect-Onset and Effect-Offset

Although, the effect-onset lack of response occipital spectrum (Figure 3F) did not exhibit an awake-alpha oscillation peak, the effect-offset lack of response occipital spectrum (Figure 4F) exhibited an awake-alpha oscillation peak, albeit lower in power compared to the response spectrum. Therefore, recovery of awake-alpha oscillation power may not sufficiently explain recovery from altered arousal states. We note that gamma oscillations were decreased in the no-response spectra compared to the response spectra during both sevoflurane effect-onset and -offset. Thus, gamma oscillations may also be essential for responsiveness to behavioral stimuli. Although decreased gamma oscillations were not clearly evident in the frontal electrodes during the no-response states, power changes in gamma frequencies (>50 Hz), which were not analyzed in this manuscript, may be associated with frontal electrodes.

### Limitations

Although the EEG recordings analyzed in this article were obtained from a high-density study, our analysis was limited to the sensor space. Therefore, we cannot make inferences on the precise brain sources of the oscillatory dynamics we describe. Future studies with EEG source localization, and intracortical recordings with temporally precise behavioral stimuli are necessary to inform more detailed mechanistic insights. Spectral leakage is associated with spectral estimation methods. Thus, our results are limited by our spectral resolution.

We conclude that decreased awake-alpha oscillation power is associated with altered arousal, and that this oscillatory dynamic may inform our understanding of large-scale brain network integration. Further, cross-frequency interactions between awake-alpha, beta and gamma oscillations may contribute to altered arousal states.

## Ethics Statement

This protocol, which was conducted at the Massachusetts General Hospital, Boston, MA, USA was approved by the Partners Human Research Committee. All volunteers gave written informed consent in accordance with the Declaration of Helsinki.

## Author Contributions

OA and ENB conceived the study. OA and PLP designed the study. OA and KJP oversaw the study. OA, KJP, EE, RV and JR acquired data. KJP, LS, LEH, EE, RI, GD, LG, OA, PLP and ENB interpreted data. KJP, LS, LEH, RI, GD, LG and OA analyzed the data and wrote the manuscript. All authors approved the final manuscript.

## Conflict of Interest Statement

The authors declare that the research was conducted in the absence of any commercial or financial relationships that could be construed as a potential conflict of interest.

## Supplementary Material

FIGURE S1 Overlay of subject level spectra for baseline awake, sevoflurane effect-onset no response and sevoflurane general anesthesia (GA). These spectra illustrate that awake-alpha power in the sevoflurane effect-onset no-response spectra were decreased compared to baseline-awake spectra. Additionally, sevoflurane-GA spectra were associated with larger slow-delta oscillation power compared to the baseline-awake and sevoflurane effect-onset no response spectra. Sevoflurane-GA spectra were also associated with decreased gamma oscillation power compared to the baseline-awake spectra.
