# Supplementary figures and images for: Lack of Responsiveness during the Onset and Offset of Sevoflurane Anesthesia Is Associated with Decreased Awake-Alpha Oscillation Power
Source: Front Syst Neurosci. 2017 May 30;11:38. doi: 10.3389/fnsys.2017.00038 (PMC5447687; doi:10.3389/fnsys.2017.00038)

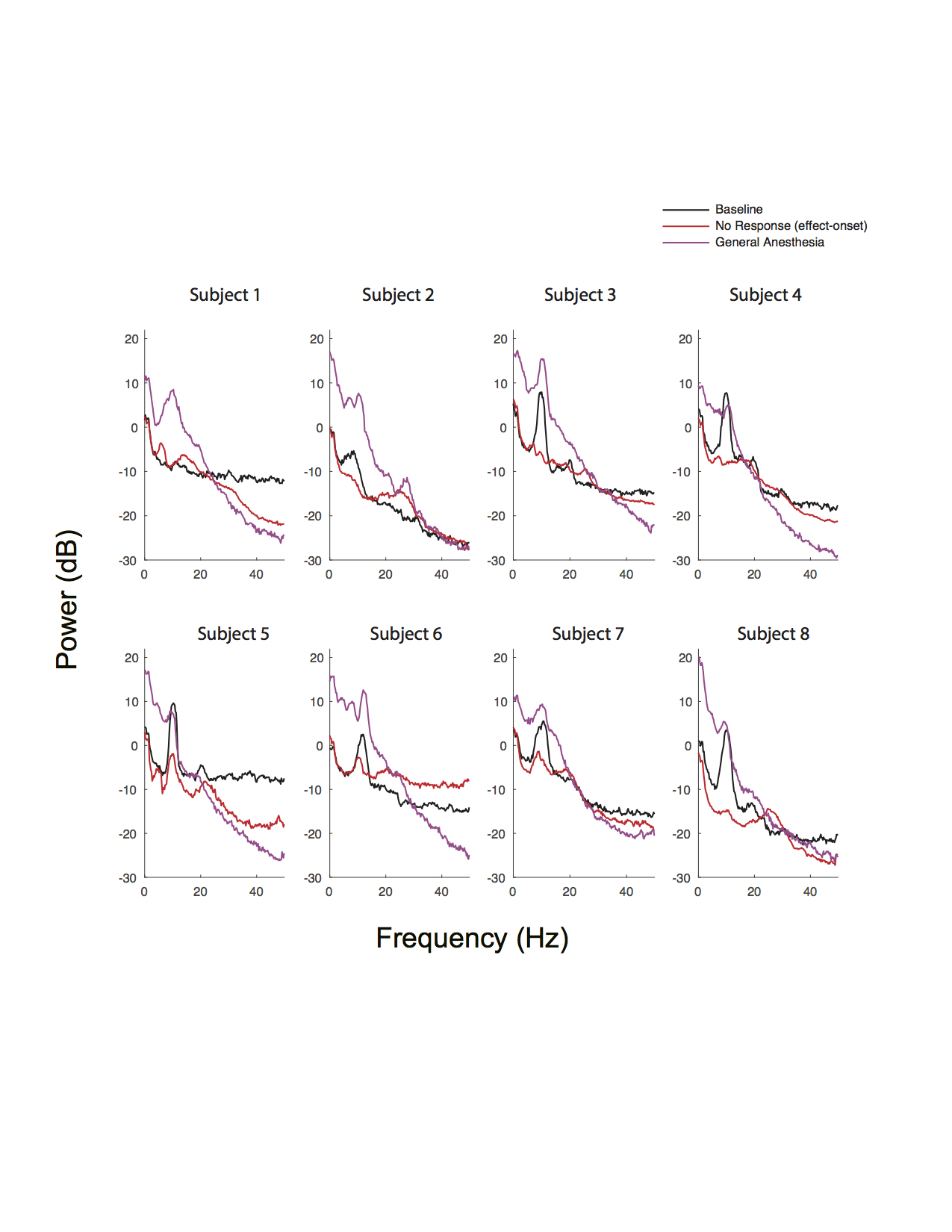

Supplement: FIGURE S1 — Overlay of subject level spectra for baseline awake, sevoflurane effect-onset no response and sevoflurane general anesthesia (GA). These spectra illustrate that awake-alpha power in the sevoflurane effect-onset no-response spectra were decreased compared to baseline-awake spectra. Additionally, sevoflurane-GA spectra were associated with larger slow-delta oscillation power compared to the baseline-awake and sevoflurane effect-onset no response spectra. Sevoflurane-GA spectra were also associated with decreased gamma oscillation power compared to the baseline-awake spectra. [file Image_1.jpeg]
